# Targeting Lumbar Spinal Neural Circuitry by Epidural Stimulation to Restore Motor Function After Spinal Cord Injury

**DOI:** 10.1007/s13311-016-0421-y

**Published:** 2016-02-02

**Authors:** Karen Minassian, W. Barry McKay, Heinrich Binder, Ursula S. Hofstoetter

**Affiliations:** Center for Medical Physics and Biomedical Engineering, Medical University of Vienna, Vienna, Austria; Hulse SCI Research Lab, Crawford Research Institute, Shepherd Center, Atlanta, GA USA; Neurological Center, Otto-Wagner-Hospital, Vienna, Austria

**Keywords:** Epidural spinal cord stimulation, Human, Motor recovery, Residual supraspinal control, Spinal circuitry, Spinal cord injury

## Abstract

Epidural spinal cord stimulation has a long history of application for improving motor control in spinal cord injury. This review focuses on its resurgence following the progress made in understanding the underlying neurophysiological mechanisms and on recent reports of its augmentative effects upon otherwise subfunctional volitional motor control. Early work revealed that the spinal circuitry involved in lower-limb motor control can be accessed by stimulating through electrodes placed epidurally over the posterior aspect of the lumbar spinal cord below a paralyzing injury. Current understanding is that such stimulation activates large-to-medium-diameter sensory fibers within the posterior roots. Those fibers then trans-synaptically activate various spinal reflex circuits and plurisegmentally organized interneuronal networks that control more complex contraction and relaxation patterns involving multiple muscles. The induced change in responsiveness of this spinal motor circuitry to any residual supraspinal input via clinically silent translesional neural connections that have survived the injury may be a likely explanation for rudimentary volitional control enabled by epidural stimulation in otherwise paralyzed muscles. Technological developments that allow dynamic control of stimulation parameters and the potential for activity-dependent beneficial plasticity may further unveil the remarkable capacity of spinal motor processing that remains even after severe spinal cord injuries.

**Electronic supplementary material:**

The online version of this article (doi:10.1007/s13311-016-0421-y) contains supplementary material, which is available to authorized users.

## Introduction

Severe spinal cord injury (SCI) is a catastrophic condition, causing disability of vital body functions below the lesion level. While contemporary clinical standards are successfully applied to deal with emergency and secondary complications, and the understanding of spinal cord biology is continuously growing, SCI still cannot be cured and the prognosis for the recovery of meaningful voluntary motor control and locomotion after a clinically complete lesion is very limited [1]. One approach to improving recovery is to reactivate the intrinsic capacity of the spared lumbar motor circuitry distal to the lesion using externally applied stimulation [2–4]. Epidural spinal cord stimulation (SCS) has been found to be particularly effective for this purpose in humans [5, 6], probably because it can provide an excitatory drive to several spinal cord segments simultaneously [7]. The first application of SCS was for the treatment of chronic intractable pain, motivated by neurophysiological studies suggesting that it was possible to inhibit input from pain fibers into the spinal cord by the stimulation of large-diameter sensory fibers [8, 9]. For the relief of diffuse pain, it seemed reasonable to stimulate the posterior columns of the spinal cord white matter, where ascending continuations of cutaneous fibers related to multiple dermatomes are compactly arranged. Norman Shealy tested this idea experimentally in 1967 by applying electrical stimulation subdurally to the posterior columns in cats [10], followed by the first human application of SCS to manage temporarily severe pain in a patient with cancer [11]. Gradually, with changing the electrode placement from a subdural to epidural location and with technological advancements, SCS became widely used [12]. The first commercially available systems used electrodes connected to implanted, radiofrequency-driven passive receivers. Later, with improved battery technology, fully implanted systems, consisting of a pulse generator with internal power source, leads, and electrodes, became available. SCS was approved by the US Food and Drug Administration in 1989 for the treatment of chronic intractable pain of the trunk or limbs and has become the most common of all neuromodulation therapies [13]. SCS has been applied off-label in many other disabling conditions, including motor disorders [14]. Here, we review the use of SCS as a method that targets the lumbar circuitry to restore motor function after severe SCI, with a focus on the underlying neurophysiology.

## Accessing the Lumbar Spinal Motor Circuitry by Epidural Stimulation

Epidural SCS has a long history of application in various motor disorders [15, 16]. It began with the serendipitous observation made in a patient with multiple sclerosis (MS) being treated for intractable back pain who regained considerable voluntary motor control with SCS [17]. Subsequent studies described improved bladder function, reduced spasticity, a feeling of lightness of the legs during movement as the primary benefits, along with increased endurance during ambulation and the recovery of some voluntary motor function with SCS [14, 18–20]. These improvements surpassed those produced by any other treatments but were not achieved in all patients [20–24], likely because of the pathophysiological complexity of MS and the varied rostrocaudal sites of SCS applied in the different studies [25].

**Fig. 1:**
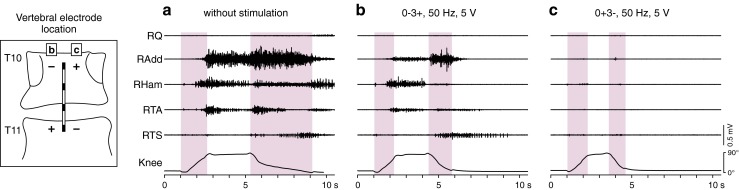
Accessing spinal circuits involved in lower-limb motor control depends on the site of epidural stimulation. Electromyographic activity elicited by tonic stretch reflex in an individual with spinal spasticity during passive hip and knee flexion-extension movements (a) without stimulation and (b, c) during 50-Hz stimulation continuously applied from either of 2 stimulation sites (rostral and caudal active cathode sites, respectively) but otherwise unchanged stimulation parameters. Intensity was set at a submotor threshold level. Near complete suppression is achieved when shifting the stimulation site from low thoracic to upper lumbar spinal cord segments (cf. [7]). Shaded backgrounds mark flexion and extension phases of the passive movement in the supine position. Q = quadriceps; Add = adductors; Ham = hamstrings; TA = tibialis anterior; TS = triceps surae of the right (R) lower limb; subject 5 in [29] with chronic motor complete, sensory incomplete spinal cord injury (American Spinal Injury Association Impairment Scale grade B, neurological level of injury: C8)

Research into the use of SCS in SCI continued, where the relationship between stimulation site and the spinal level and severity of the lesion were better defined [26]. Stimulation located rostral to the lesion was determined to have a less-than-satisfactory impact on spasticity [22], while stimulation caudal to the lesion markedly suppressed it when delivered to the spinal segmental levels related to the distribution of the spasticity [27]. Dimitrijevic et al. [28] described that, of the patients with cervical lesions and electrodes placed just below the lesion, those with partially preserved motor and sensory function obtained marked effects, while the ones with complete lesions showed no effect on leg spasticity. Control of leg spasticity in patients with complete lesions was best achieved when electrodes were placed over the posterior structures of the lumbar spinal cord [28]. Similarly, Pinter et al. [29] demonstrated considerable suppression of severe leg spasticity in all patients with chronic SCI studied, when stimulation targeted the upper lumbar spinal cord (Fig. 1). Hence, the effects of SCS depended on the individual SCI profile, and the rostrocaudal location of the electrodes relative to the lesion and to the lumbar segments of the spinal cord [28].

The interpretation of these earlier results was facilitated by increasing knowledge of the neural structures being electrically stimulated. At therapeutic intensities for the management of pain or spasticity (generally 0.5–5 V with an impedance of 300–1000 Ω for a bipolar electrode configuration), large-to-medium-diameter sensory fibers within the posterior roots [30], or their branches within the posterior columns of the spinal cord white matter [31], are stimulated, depending on the segmental anatomy and its functional integrity at the stimulation site, and on the stimulation intensity and pulse width. At lumbosacral segmental levels, posterior roots are a prominent component of the intrathecal neuronal structures [32] and are the primary targets for lumbar SCS [7]. They contain the wide range of sensory fibers arriving from the muscles, tendons, joints, and cutaneous tissues of the hips and legs. The posterior columns are largely made up of the rostral continuation of the cutaneous root fibers, because the primary afferents from muscles and joints of the lower limbs leave the posterior columns and ascend through secondary systems [33]. Spinal interneurons and motoneurons are not electrically activated but can be trans-synaptically recruited through the stimulation of the sensory fibers [34, 35].

In people with incomplete cervical lesions, SCS below the lesion was postulated to act through spinal and brainstem levels [28, 36]. It was thought that orthodromic conduction evoked within the posterior columns may have increased descending activation of spinal inhibitory circuitry through brainstem–spinal cord loops [28]. In addition, the antidromic activation of the posterior-column fibers could have modulated the activity of the lumbar segmental circuitry involved in the regulation of afferent inputs and of motoneuronal excitability [37]. In the individuals with complete cervical SCI, the posterior-column fibers were either functionally not intact at the stimulation site, or the effects would have required the stimulation of fiber types originating in the legs that are not present in the posterior columns at such distance from the lumbar spinal cord. With electrodes over the lumbar spinal cord, however, the stimulation accessed the local spinal circuitry via the posterior root afferents [29]. SCS applied to the same site, yet with frequencies lower than the range of 50–100 Hz normally used for spasticity control [29], was the key to novel studies examining the motor capacity and processing characteristics of the lumbar spinal cord [5].

## Motor Capacity of the Lumbosacral Spinal Cord Circuitry Below a Paralyzing Injury

The finding by Dimitrijevic et al. [5] that the lumbar spinal circuitry has the capacity to produce multisegmentally coordinated output to lower-limb muscles in people with no or minimal supraspinal influence initiated a series of studies on the underlying neurophysiological mechanisms that can be set into action by SCS. One approach to examining the complexity of these mechanisms is to explore the effects of different SCS frequencies.

**Fig. 2:**
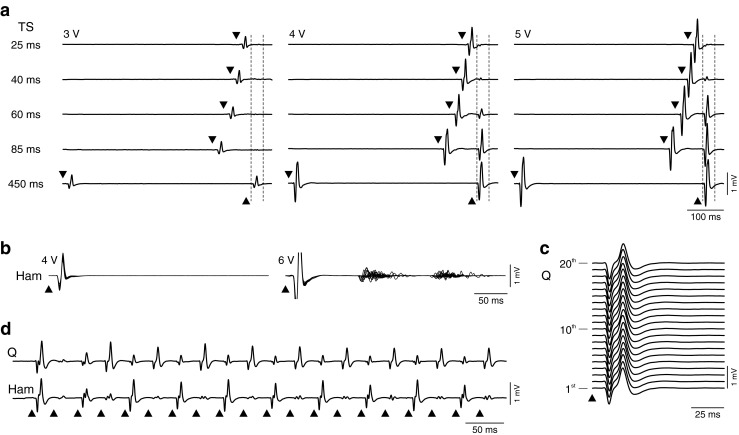
Spinal cord stimulation (SCS) activates spinal reflex circuitry. (a) Responses to paired pulses at conditioning test intervals and stimulation intensities as indicated. Stimulus application is marked by black arrowheads; vertical dashed lines show time windows of second responses. The reflex nature of the responses is revealed by the depression with decreasing interstimulus intervals. The degree of recovery increases with the size of the unconditioned reflex. Triceps surae (TS) of an individual with complete spinal cord injury (SCI) [subject 1 in [30], classified as American Spinal Injury Association Impairment Scale (AIS) grade A; neurological level of injury: C7). (b) Response types to single stimulus pulses. Each graph shows 20 superimposed responses of hamstrings (Ham) from a person with motor complete, sensory incomplete SCI (subject 6 in [51], AIS grade B; neurological level: T8). Each pulse evoked a short-latency posterior root-muscle (PRM) reflex, without (left) or with long-latency, polysynaptic responses. (c) Response behavior during repetitive stimulation. Monosynaptic PRM reflexes evoked by 2-Hz SCS. First 20 responses of quadriceps (Q) of an individual with complete SCI (subject 4 in [45], AIS grade A; neurological level: C5). Note the repeatability of the response waveform. (d) With increasing frequency, neural circuits involved in the regulation of spinal reflex activity are integrated, as reflected here by the patterns of PRM reflex modulation; 16 Hz, same subject as in (c). Note the reciprocal relationship between Q and Ham muscles

Each stimulus pulse essentially activates motoneurons through monosynaptic connections, to evoke a posterior root-muscle (PRM) reflex when stimulus strength is adequate [34, 38]. This PRM reflex probably occurs because the afferent volley produced is highly synchronized [7], and because of the large excitatory postsynaptic potentials evoked by group Ia afferents in the motoneurons [39]. Owing to the proximity of posterior roots of several lumbar and upper sacral segments to the stimulating epidural electrode, PRM reflexes are normally elicited in many leg muscles bilaterally by a single pulse. When paired pulses are applied, the second PRM reflex is depressed with decreasing interstimulus intervals (Fig. 2a) [34], which is a hallmark property of monosynaptic reflexes [40]. Similarly, paired pulses of SCS in intact adult rats at interstimulus intervals of 10–500 ms led to a significant decrease of the reflex components evoked by the second pulse [35, 41], and complementary pharmacological experiments more directly verified the monosynaptic reflex component [35]. The short-latency PRM reflexes can be followed by long-latency reflexes or longer-lasting after-discharges (Fig. 2b), with onsets delayed by more than 50 ms with respect to the monosynaptic latency [42, 43]. These are most likely polysynaptic reflexes, either evoked by sensory inputs of different modality [43], or reflecting an increased responsiveness of the interneuronal circuitry associated with spasticity [44].

When SCS is applied continuously, the simplest response type is the stimulus-time locked monosynaptic PRM reflex evoked at 2 Hz (typically the lowest available stimulation frequency in the fully implanted system) with no modulation or depression within the series of responses (Fig. 2c) [34, 45]. With increasing stimulation frequencies, there is a general decrease in the motor output produced [5, 42, 46]. Yet, within distinct frequency ranges, the interneuronal circuitry can generate temporarily stable output patterns. With stimulation around 16 Hz, patterns emerge with the consecutively elicited PRM reflexes alternating between large and small amplitude waveforms (Fig. 2d) [45]. These patterns are most likely due to interactions when the inputs evoked by a given pulse arrive at the spinal circuitry and motoneurons before the cessation of neural activity initiated by previous stimuli [45]. Potential mechanisms with appropriate duration include presynaptic inhibition and postactivation depression, as well as recurrent inhibition and facilitation [40], implying that SCS trans-synaptically activates interneuronal circuits involved in the regulation of afferent input and motoneuronal excitability.

SCS at 22–50 Hz can generate locomotor-like electromyographic (EMG) activity and induce involuntary leg flexion-extension movements in individuals with chronic, motor complete SCI lying supine [5, 47]. Thus, in the absence of a volitional motor task or gait-specific peripheral feedback, the human lumbar spinal circuitry can generate activity resembling the output of a central pattern generator [48] in response to tonic input. At these stimulation frequencies, the EMG outputs are a series of PRM reflexes with burst-like amplitude modulation superimposed [7, 34], suggesting that the posterior-root input activated pattern-generating networks, which, in turn, modified and coordinated the PRM reflex activity at multiple segmental levels. While the SCS-induced rhythmic activity consists of PRM reflexes with monosynaptic latencies during the extension-like phases, these responses could be completely suppressed and replaced by oligosynaptic reflexes during the flexion-like phases, with onset latencies delayed by approximately 10 ms [7, 34, 49]. The nature of these responses is not clear, but they could be related to the oligosynaptic reflexes that can be evoked in addition to the monosynaptic ones by SCS in intact or spinal adult rats, which were suggested to involve group II reflex circuits [35], to be mediated by spinal circuitry associated with the flexor reflex [41], or to reflect the contribution of spinal locomotor networks to the motor output [50].

**Fig. 3:**
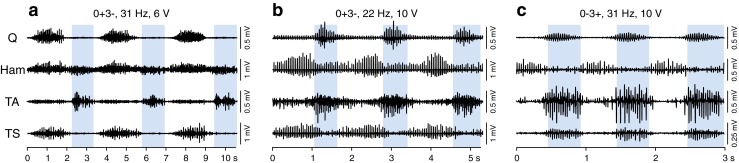
Spinal cord stimulation (SCS) activates spinal pattern generating networks. Rhythmic electromyographic activity with different multimuscle activation patterns and burst frequencies evoked by tonic epidural SCS in individuals with motor complete spinal cord injury lying supine. Three complete cycles are displayed for each example. The patterns include combinations of co- or alternating activation of muscles within flexor- (shaded background) and extensor-like phases. (a) Patient 4 in [51], classified as American Spinal Injury Association Impairment Scale (AIS) grade B (neurological level of injury: C8); (b) patient 3 in [51], AIS grade B (neurological level: T9); (c) patient 7 in [30], AIS grade A (neurological level: T7); active electrode combinations of the 4-contact linear array and stimulation parameters as indicated. Q = quadriceps; Ham = hamstrings; TA = tibialis anterior; TS = triceps surae

A recent study explored the capacity of the functionally isolated human lumbar spinal cord to produce a wide range of coordinated rhythmic multimuscle activation patterns under tonic SCS (Fig. 3). Statistical analysis revealed that these patterns are based on flexible combinations of a small number of activation timing profiles, each probably reflecting the neural drives of spinal burst generators [51]. Most of the rhythmic EMG samples were generated with an epidural cathode position over the L2–L4 spinal cord segments, suggesting that the upper lumbar posterior-root fibers have specifically effective projections onto the locomotor circuitry or that key elements of this circuitry are distributed within these segments [52].

In summary, the motor output generated by lumbar SCS is the integral of repetitively activated mono-, oligo-, and polysynaptic reflex circuits, as well as of the operation of a more complex, plurisegmentally organized network recruited by the tonic nature of the input [7, 53]. The network’s activity modulates the series of evoked reflexes by pre- and postsynaptic actions [50]. This interneuronal network can be organized with certain stimulation frequency ranges to control contraction and relaxation of a muscle as part of a variety of motor patterns [51]. Apart from the SCS-provided input, it is likely that the network’s output also depends on its state of excitability. This was shown for the circuits underlying the tendon tap reflex in spastic conditions [44], as well as for more complex ones involved in the generation of rhythmic activity in paralyzed leg muscles during assisted treadmill stepping [4, 54, 55]. Preliminary findings indicate that an increased state of excitability in the lumbar circuits increases the probability that rhythmic activity will be generated when SCS is applied at 22–50 Hz [42].

## Supraspinal Influence on Spinal Circuits Below a Paralyzing Lesion

Voluntary leg movements are largely controlled through the activation and regulation of spinal interneurons [56], many of which are parts of reflex and locomotor circuitry and receive convergent control from supraspinal centers, axons originating in the spinal cord, and sensory afferents [57, 58]. Supraspinal control can thus be expressed through facilitation and suppression of the excitability and operation of the spinal interneuronal systems that finally provide integrated input to the motoneurons.

Postmortem anatomical studies have revealed that residual white matter crossing the lesion is a common finding even in those individuals with an SCI classified as clinically complete, that is, without voluntary movement and sensation below the injury [59–61]. Conduction along such preserved fibers and their functional role in conveying intentional motor control must be clearly compromised, yet may provide for some subclinical brain influence over spinal excitability caudal to the lesion [62]. Neurophysiological evidence of such residual influence was found to be present in a majority of clinically complete SCI individuals, which led to the definition of the discomplete SCI [63–65].

**Fig. 4:**
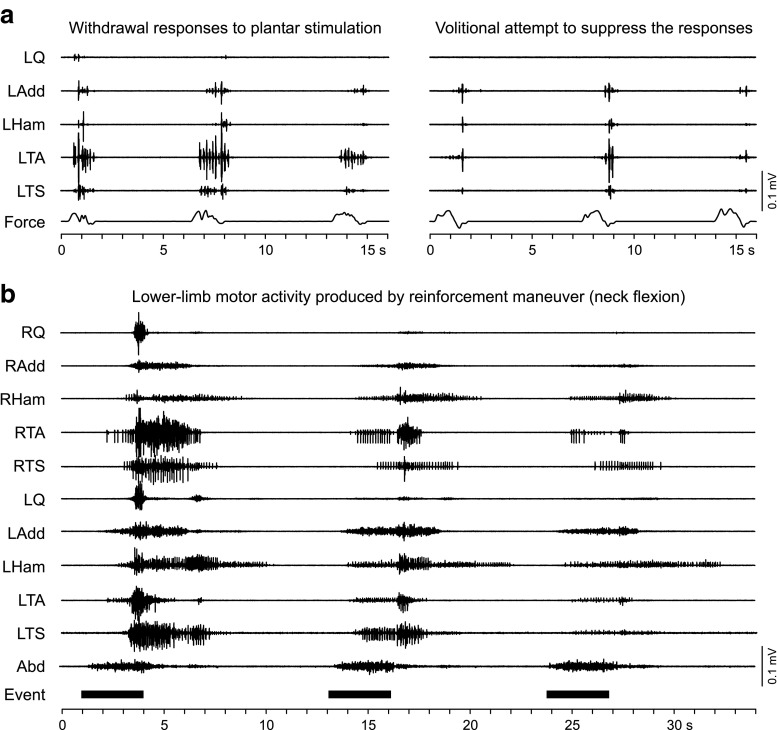
Neurophysiological evidence for residual translesional inhibitory and excitatory influence in individuals with clinically motor complete spinal cord injury. (a) Withdrawal responses evoked by stroking the left foot sole and its voluntary suppression (patient 4 in [51], classified as American Spinal Injury Association Impairment Scale (AIS) grade B; neurological level of injury: C8). The bottom trace (Force) is a strain gauge registering the force exerted as the stimulating probe was drawn along the plantar surface. (b) Delayed and widespread activation of paralyzed lower limb muscles by forceful isometric neck flexion performed with the patient in the supine position (patient 3 in [51], AIS grade B; neurological level: T9). Three repetitions are denoted by the event marker, and by the electromyographic activity in the abdominal (Abd) muscles. Note the supraspinally induced non-specific activation of muscles that could not be activated by volitional single- or multijoint motor tasks. Q = quadriceps; Add = adductors; Ham = hamstrings; TA = tibialis anterior; TS = triceps surae

Descending volitional activation of spinal inhibitory function in paralyzed individuals was demonstrated taking the form of intentional suppression of spinal reflex responsiveness below the lesion level (Fig. 4a) [64–66]. Indirect yet intentionally initiated supraspinal activation of motor units in paralyzed legs can be revealed by reinforcement maneuvers, that is, by forceful, voluntary activation of nonparalyzed muscles above the lesion (Fig. 4b) [64, 65, 67]. Two types of responses to such reinforcement maneuvers were distinguished, one appearing only in a few muscles and with an onset latency of 0.8–1.2 s, and another one appearing in multiple muscles with a latency of 2–3 s [67]. The long response latencies suggest transmission through a slowly conducting residual descending system, with probable additional delay for activation of a plurisegmental interneuronal network being responsible for the second, later and more widespread response type [67]. The reinforcement maneuvers not only elevate spinal cord excitability below the lesion level time-related to a motor task, but the generalized activation of multiple muscles can also evoke synergistic multijoint flexion movements in paralyzed limbs [54, 68], yet amplitude or duration of these movements cannot be volitionally controlled [67].

When encouraged to perform a purposeful movement of the paralyzed limbs, some individuals with discomplete SCI are able to generate task-specific EMG activity. Instructed to flex hip and knee joints or, focally, the ankle joint, approximately 10 % of the individuals with discomplete SCI generated traces of motor unit activity, yet not strong enough or insufficiently coordinated to elicit a visible contraction or movement [64, 65].

In conclusion, there is a high likelihood that clinically silent translesional neural connections can survive even a severe SCI. That these neural pathways could provide a basis for recovery through enhancement of residual functions by future treatment approaches was already predicted in early anatomical and physiological studies [60, 67].

## Interaction of Subclinical, Volitional Descending Input with Epidural SCS

Clinically viewed, many cases of augmented function, including improvement in motor strength and voluntary motor function, were reported in the early applications of SCS in MS and SCI [18, 23, 27, 28, 69]. A detailed observation of volitional movement enabled by SCS was reported in a patient with a C5-incomplete SCI treated for severe lower-limb spasticity [70]. The stimulating cathode was at the T1–T2 level. Stimulation frequencies were 30–100 Hz and intensities set to induce paraesthesias in the lower extremities without causing muscle activation. Long-term control of spasticity was achieved soon after implantation. In an 8-month follow-up, a few seconds after SCS was turned on, the patient could contract and relax the otherwise paralyzed left quadriceps during ongoing stimulation strongly enough to completely extend the knee against gravity. This ability stopped immediately after stimulation was turned off. The stimulation had clearly activated cutaneous posterior-column fibers related to the L2–S2 dermatomes. Antidromic activity conducted along these fibers could then reach the lumbar spinal cord circuitry and increase its responsiveness to otherwise ineffective volitional descending input [37].

**Fig. 5:**
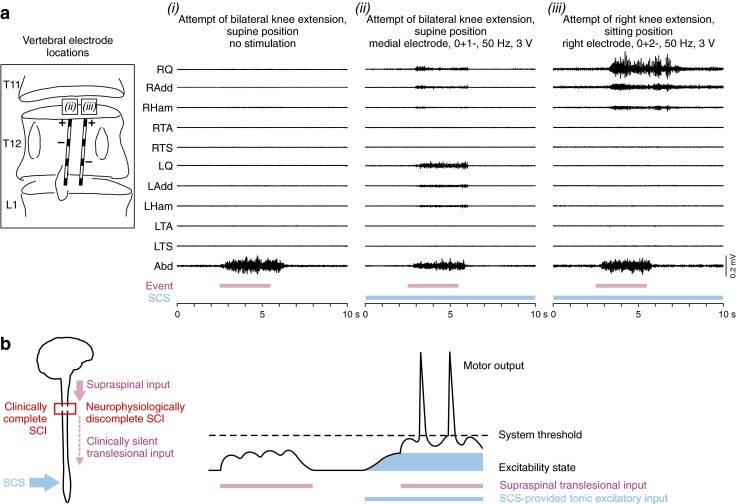
Task-related motor output during attempts to voluntarily move paralyzed lower limbs enabled by spinal cord stimulation (SCS). (a) Electromyographic (EMG) activity during attempted bilateral knee extension while supine *(i)* without and *(ii)* with SCS, and *(iii)* right knee extension in the sitting position with SCS, as illustrated on the left. Stimulation was applied at the motor threshold of right quadriceps (RQ) and right adductor (RAdd) in *(iii)*, and was otherwise below the motor threshold. Purple bars indicate periods of voluntary attempts; the EMG activity in the abdominal (Abd) muscles coincides with the patient’s effort. Blue bars indicate SCS application. SCS in itself did not induce muscle contraction or movement at the intensities applied. Q = quadriceps; Add = adductors; Ham = hamstrings; TA = tibialis anterior; TS = triceps surae of right (R) and left (L) side. Unpublished results of patient 1 in [29], classified as American Spinal Injury Association Impairment Scale (AIS) grade B (neurological level of injury: C6). (b) Simplified sketch of a possible explanation for the immediate enabling effect of SCS. SCS-provided excitatory input moves the central state of excitability closer to threshold and enables an otherwise ineffective supraspinal input to generate motor output. Sketch on the right side is adapted, with permission, from Fig. 35–8 in [87]

A similar case involving a patient with a chronic, clinically motor complete, and sensory incomplete SCI is presented in Fig. 5(a). Without stimulation, the patient could neither generate muscle contractions nor task-related EMG activity in the lower limbs. In the supine position during 50-Hz lumbar SCS, the patient activated all studied thigh muscle groups when prompted by the examiner to extend the legs. In the sitting position, the attempt of voluntary right knee extension activated the right thigh muscles strongly enough to extend the leg, with a delay of approximately 0.8 s relative to the auditory command cue, suggesting neural transmission and processing through a slow-conducting system. Summation processes of limited translesional input with the lumbar circuit’s excitability enhanced by the tonic input through the posterior root afferents are a likely explanation for the immediate enabling effect of SCS (Fig 5b).

A recent single-case study considerably increased the interest in lumbar SCS as a neuroaugmentative intervention [6]. There, a patient with a motor complete, sensory incomplete SCI noticed, 7 months after implantation, that the stimulation enabled some rudimentary, intentionally induced movements of the paralyzed legs in the supine position. A succeeding study was then specifically designed to investigate this enabling effect of SCS [71]. All 4 individuals with chronic, clinically motor complete SCI studied (of whom 2 had preserved sensation, including the patient from [6]) could induce movement while supine with stimulation intensities close to or at the motor threshold level and a stimulation frequency of 25 Hz or 30 Hz. In the additional 3 individuals, this was possible during the first experimental session. Enabled movements included hip and knee flexion, ankle dorsiflexion, and toe extension, and could be timed to visual or auditory cues. Two patients were able to generate graded levels of force. With continued home training, one patient temporarily maintained the ability to perform leg flexion even after SCS was turned off. It is likely that different descending systems and spinal mechanisms were involved individually, as well as between motor tasks in [71]. Hip and knee flexion is a task that requires intensive voluntary effort accompanied by isometric contractions of neck, trunk, and arm muscles, and could result in a “reinforcement-type” activation of the legs [65, 68], augmented by the ongoing SCS. Yet, isolated ankle or toe movement and the ability of controlling graded activity suggest that SCS had enabled more specific motor control mechanisms [71]. The immediate enabling effect of the stimulation in the 3 additional participants implied that the underlying residual descending connections had existed since the time of their injury and could utilize the increased excitability of the lumbar circuitry brought by SCS to generate motor output. Earlier studies had already indicated that residual supraspinal input may require a critical level of base excitability within the lumbar spinal circuits to produce motor output, as the lowest percentages of individuals with discomplete SCI were identified in studies with the highest percentage of patients taking antispasticity medication [64, 65, 67]. The requirement of SCS frequencies of 25 Hz or 30 Hz may further suggest that the tonic drive also facilitated the inherent capacity of the lumbar circuitry to produce synergistic flexion or extension output [34, 51].

The long periods of stimulation combined with intensive active training additionally raised the question of whether they could have resulted in improved functional, translesional connections with the lumbar circuitry [6, 71], as demonstrated in adult rats after a paralyzing SCI [72]. The rats with staggered lateral hemisection injuries, suspended by a robotic-controlled harness, were encouraged to use actively the paralyzed hindlimbs to walk overground toward a food reward, while SCS and monoamine agonists were applied to elevate the physiological state of the lumbosacral locomotor circuitry. After a few weeks of training, they could initiate and sustain full weight-bearing bipedal locomotion during the stimulation. Anatomical examinations identified a remodeling of descending axonal projections and relays within the spared tissue bridge between the 2 hemisections, as well as within the segmental levels above the lesions and brainstem motor areas. Electrophysiologically, supraspinal volleys to hindlimb motor pools through slow-conducting pathways could be verified, suggested to be transmitted through the new contacts with relay neurons that bypassed the injury.

## Interaction of Step-induced Feedback with Epidural SCS

Sensory feedback input from the legs to the lumbosacral spinal cord induced by externally imposed stepping motions on a treadmill can generate step-phase synchronized EMG activity in individuals with SCI who are not able to produce similar motor patterns voluntarily [54, 55, 73]. Yet, in individuals with motor complete SCI, such input recruits only a few muscles, or the produced patterns are inappropriate in amplitude or timing to result in unassisted movements [74]. It is thought that, being deprived of supraspinal excitatory support, an appropriate state of activation of the lumbar pattern generating networks cannot be sustained by cyclic peripheral feedback alone [4, 25]. Indeed, multisegmental excitatory drive provided by SCS at 20–50 Hz in 2 motor and sensory complete SCI individuals during assisted treadmill stepping immediately enhanced rhythmic motor output over purely assisted stepping [75]. With SCS intensity close to, yet below, the threshold for eliciting PRM reflexes in a given muscle, the rhythmic activity produced by assisted stepping alone was amplified and additional muscles were activated. Stronger stimulation generated tonic motor output during supported standing, but this activity became rhythmic as soon as stepping was imposed by the therapists. Thus, SCS-provided tonic excitatory input and phasic feedback from the legs, both carried by similar afferent fiber populations [76], are integrated by the lumbar circuitry in the generation of rhythmic motor outputs. Even after intensive locomotor therapy, the results achieved in the patient from the case study of Harkema et al. [6], in whom assisted stepping was combined with 30–40 Hz SCS, were very similar to the immediate effects of SCS described previously [75]. In their succeeding study however [71], 2 patients with SCI could further augment the EMG activity as produced by assisted treadmill stepping and epidural stimulation, when consciously thinking about moving the legs. While independent stepping was not yet achieved in individuals with an SCI clinically classified as motor complete, treadmill training with voluntary contribution during an SCS-enhanced physiological state of the locomotor circuitry may lead to unexpected levels of recovery in a select group of patients. In rats after a paralyzing injury, automated treadmill training with electrical and chemical stimulation of the lumbosacral circuitry failed to promote translesional plasticity and recovery of hindlimb overground locomotion with trunk support, whereas overground training with the rats encouraged to use voluntarily the paralyzed hindlimbs led to the formation of indirect descending pathways and the ability to step toward a target [72]. Further, a recent study in mutant mice demonstrated that selective degeneration of muscle spindles severely limited the formation of compensatory translesional connections to deprived circuits below the injury level for locomotor recovery after incomplete SCI, while wildtype mice spontaneously recovered basic locomotor function [77]. Thus, the translesional plasticity after SCI was suggested to have been steered by activity within the spinal circuitry accessed by muscle spindle fibers [77], that is, by structures that are also engaged by SCS [34, 35]. Although such animal studies must be carefully interpreted, given the known differences in the injury models and interspecies differences in neuroanatomy and its ability to regenerate after injury, they provide important insights into potential mechanisms of SCS that could partially restore descending motor control after SCI in humans. The patient population with motor incomplete SCI who remain wheelchair bound after standard-of-care rehabilitation and intensive locomotor training may most profoundly benefit from currently available SCS systems used in combination with active rehabilitation training programs [78, 79].

## Conclusion

New therapeutic paradigms for use in neurological disorders have evolved out of engineering advancements coupled with an increasing understanding of the underlying physiology [12]. In recent decades, progress has been made in understanding the mechanisms by which the central nervous system controls movement in humans. Evidence has accumulated that, at the spinal level, human locomotion is controlled by neural circuitry similar to that of other walking vertebrates [40, 80]. Lessons have been learned from experimental studies that the enhancement of the functional state of the spinal motor circuitry induced by neuromodulatory techniques is likely to be essential for the development of new rehabilitation paradigms to improve therapeutic outcomes after severe SCI [2, 3, 71, 72]. However, engineering efforts in the development of SCS systems have concentrated on their optimization for the treatment of chronic pain. The same systems have been also used in motor disorders where available electrode designs and the lack of flexibility in the control of the parameters are obvious technological limitations. In the activation of the lumbar motor circuitry, it can be suggested that tonic SCS partially compensates for the loss of excitatory drive from the brainstem in paralysis due to SCI [4]. Obviously, for the control of locomotion such stimulation is rather crude, as descending inputs have multiple roles, including cycle-to-cycle regulation of locomotor activity [81]. Hence, a future technological breakthrough could include the closed-loop, real-time control of task- and phase-specific parameters of epidural SCS [53, 82]. The availability of noninvasive methods of SCS that activate similar input structures to the lumbar circuitry may add to the crucial understanding of which physiological lesion profiles within the clinical categories of SCI will respond to such interventions [83–86]. Finally, it should be noted that secondary beneficial physiological effects have been reported with epidural SCS, including improved peripheral blood flow, bladder and bowel function, sexual function, and production of sweating below the lesion level, which are of high priority for individuals with SCI [1, 6, 26, 27].

## Electronic supplementary material

Below is the link to the electronic supplementary material.

ESM 1 (PDF 1224 kb)
